# Genomic Sequence of *Klebsiella pneumoniae* IIEMP-3, a Vitamin B_12_-Producing Strain from Indonesian Tempeh

**DOI:** 10.1128/genomeA.01724-15

**Published:** 2016-02-25

**Authors:** Adi Yulandi, Febri Gunawan Sugiokto, Febrilina, Antonius Suwanto

**Affiliations:** a Faculty of Biotechnology, Atma Jaya Catholic University of Indonesia, Jakarta, Indonesia; b Department of Biology, Faculty of Science and Mathematics, Bogor Agricultural University, Bogor, Indonesia

## Abstract

*Klebsiella pneumoniae* strain IIEMP-3, isolated from Indonesian tempeh, is a vitamin B_12_-producing strain that exhibited a different genetic profile from pathogenic isolates. Here we report the draft genome sequence of strain IIEMP-3, which may provide insights on the nature of fermentation, nutrition, and immunological function of Indonesian tempeh.

## GENOME ANNOUNCEMENT

The presence of *Klebsiella pneumoniae* in Indonesian tempeh is intriguing since it produces vitamin B_12_ in tempeh (1–3), while many strains of this species were known as opportunistic pathogens (4). *Klebsiella pneumoniae* is well known for causing pneumonia, an acute infection that attacks alveoli (5) and causes liver abscess in human (6).

Previous molecular profiling employing the Enterobacterial Repetitive Intergenic Consensus PCR (ERIC-PCR) method showed that *K. pneumoniae* isolates from tempeh exhibited different genetic profiles from those of isolates pathogenic to humans (*K. pneumoniae* ATCC BAA-2146, *K. pneumoniae* subsp. *pneumoniae* ATCC 10031, *K. pneumoniae* ATCC 35657, and *K. pneumoniae* FK isolated from a patient with pneumonia) (7).

The nucleotide sequence of *K. pneumoniae* strain IIEMP-3 was determined employing a HiSeq 2000 platform (Illumina, San Diego, CA, USA) through a commercial service provider (Macrogen, Seoul, South Korea). A 350-bp paired-end library was constructed and sequenced, producing 10 million paired-end reads with read lengths of 100 bp.

*De novo* assembly was processed using Velvet version 1.2.07 (8) with k-mer length 63 and no scaffolding option. A scaffolding process using SSPACE (9) resulted in a 350-bp insert with an error of 0.25. The scaffolding result was fixed by ICORN (10) with 5 times iteration and evaluated by REAPR (11) with aggressive breaking; as many as 51 contigs were generated from this workflow. The longest contig achieved is 827,134 bp and total bases were 5,3262,779 bp.

Genome annotation was performed employing the NCBI Prokaryotic Genome Annotation Pipeline (http://www.ncbi.nlm.nih.gov/genome/annotation_prok). A total of 5,285 genes, 5,096 coding sequences (CDSs), 106 pseudogenes, 5 rRNAs, 77 tRNAs, and 1 noncoding RNA (ncRNAs) were identified.

### Nucleotide sequence accession number.

The genome sequence of *Klebsiella pneumoniae* IIEMP-3 has been deposited in GenBank under the accession number LMAP00000000.
